# Leaf-residing *Methylobacterium* species fix nitrogen and promote biomass and seed production in *Jatropha curcas*

**DOI:** 10.1186/s13068-015-0404-y

**Published:** 2015-12-21

**Authors:** Munusamy Madhaiyan, Tan Hian Hwee Alex, Si Te Ngoh, Bharath Prithiviraj, Lianghui Ji

**Affiliations:** Biomaterials and Biocatalysts Group, Temasek Life Sciences Laboratory, 1 Research Link, National University of Singapore, Singapore, 117604 Singapore; Plant Biology Division, The Samuel Roberts Noble Foundation, Ardmore, OK 73401 USA

**Keywords:** Culturable endophyte, Nitrogen fixation, *Methylobacterium*, *Jatropha curcas* L., Biofuel

## Abstract

**Background:**

*Jatropha curcas* L. (Jatropha) is a potential biodiesel crop that can be cultivated on marginal land because of its strong tolerance to drought and low soil nutrient content. However, seed yield remains low. To enhance the commercial viability and green index of Jatropha biofuel, a systemic and coordinated approach must be adopted to improve seed oil and biomass productivity. Here, we present our investigations on the Jatropha-associated nitrogen-fixing bacteria with an aim to understand and exploit the unique biology of this plant from the perspective of plant–microbe interactions.

**Results:**

An analysis of 1017 endophytic bacterial isolates derived from different parts of Jatropha revealed that diazotrophs were abundant and diversely distributed into five classes belonging to *α, β, γ*-*Proteobacteria,Actinobacteria* and *Firmicutes.Methylobacterium* species accounted for 69.1 % of endophytic bacterial isolates in leaves and surprisingly, 30.2 % which were able to fix nitrogen that inhabit in leaves. Among the *Methylobacterium* isolates, strain L2-4 was characterized in detail. Phylogenetically, strain L2-4 is closely related to *M. radiotolerans* and showed strong molybdenum-iron dependent acetylene reduction (AR) activity in vitro and in planta. Foliar spray of L2-4 led to successful colonization on both leaf surface and in internal tissues of systemic leaves and significantly improved plant height, leaf number, chlorophyll content and stem volume. Importantly, seed production was improved by 222.2 and 96.3 % in plants potted in sterilized and non-sterilized soil, respectively. Seed yield increase was associated with an increase in female–male flower ratio.

**Conclusion:**

The ability of *Methylobacterium* to fix nitrogen and colonize leaf tissues serves as an important trait for Jatropha. This bacteria–plant interaction may significantly contribute to Jatropha’s tolerance to low soil nutrient content. Strain L2-4 opens a new possibility to improve plant’s nitrogen supply from the leaves and may be exploited to significantly improve the productivity and Green Index of Jatropha biofuel.

**Electronic supplementary material:**

The online version of this article (doi:10.1186/s13068-015-0404-y) contains supplementary material, which is available to authorized users.

## Background

*Jatropha curcas* L. (Jatropha) is a woody perennial, drought-tolerant shrub belonging to *Euphorbiaceae* and is widely distributed in tropical and subtropical regions. Jatropha seeds contain high level of triacylglyceride with a fatty composition well suited for biodiesel production [1]. Jatropha is resistant to drought, able to thrive on marginal land under climate and soil conditions that are unsuitable for food crop plantation [2–5]. In addition to sequestrating CO_2_ and reducing the world’s reliance on fossil fuel, Jatropha helps control soil erosion [6] and detoxify polluted soil [7–9]. As a wild plant, however, Jatropha seed and oil productivity remains low, particularly when chemical fertilizer input is limited. Apart from breeding programs for high-yielding Jatropha varieties [10–12], agronomical practices, such as the application of inorganic fertilizer [13] and plant growth regulators, have also been reported to improve seed yield [14, 15]. As Jatropha is targeted to plant on marginal soil with low nutrient levels, fertilizer requirement would be higher than other crops. This would significantly affect the commercial viability of Jatropha and offset the Green Index of Jatropha biofuel.

It has been increasingly realized that plants form close association with a large population of diverse bacteria, which are either loosely associated with roots (rhizosphere bacteria), actively colonizing internal plants tissues (endophyte) and leaf surfaces (epiphyte) [16–21]. Plants often benefit from such interactions because of nitrogen fixation; production of plant growth hormones, such as auxin, cytokinin and gibberellin; delayed senescence through suppression of ethylene biosynthesis by secreting 1-aminocyclopropane-1-carboxylate (ACC) deaminase; alteration of sugar sensing mechanisms [22–24] and inhibiting pathogen attacks through production of hydrolytic enzymes [25], competition for space and nutrients [26, 27], and induction of systemic defence mechanisms [28–30]. Bacterial inoculations improved growth and development of switchgrass seedlings, significantly stimulated plant growth, and tiller number on the low fertility soil, and enhanced biomass accumulation on both poor and rich soils, with more effective stimulation of plant growth in low fertility soil than in high fertility soil [31]. Our previous study also showed that *Kosakonia* species suitable for limited N-content soil and significantly promoted growth and seed yield of Jatropha [32]. Here, we present an investigation on the diversity of culturable endophytic bacteria of Jatropha and a detailed study on the role of a novel leaf-colonizing diazotroph, *Methylobacterium* sp. strain L2-4, on Jatropha biomass and seed production.

## Results and discussion

### Culturable endophytic bacterial density in Jatropha tissues

We sampled Jatropha root, stem and leaf tissues from three different germplasm accessions. Endophytic bacterial colonies were established on six different solid media after thorough surface sterilization of the plant tissues. As expected, endophytic bacterial densities varied amongst tissue types and culture media employed. The highest density was seen in roots, followed by stems while leaves showed the lowest density irrespective of the isolation media used (Additional file 1: Figure S1). Similar to previous findings [33, 34], use of complete media, such as KB media and Medium 869, resulted in higher endophyte density while synthetic media, such as AMS with methanol as carbon source, Nfb or BAz media with malic acid or azelaic acid as the carbon source, yielded lower densities (Additional file 1: Figure S1). Canonical discriminant analysis (CDA) of the combined population data of 3 germplasm accessions showed that the origin of plant tissues and media employed for the isolation formed distinct groups (Additional file 2: Figure S2). These results suggest that the distribution of endophytic population vary within different parts of Jatropha and the bacterial population will be ill-presented if the investigation relies on a single medium.

Given the large endophytic bacterial population found in various types of tissues, we focused our studies on selected representatives of culturable species, which were selected randomly according to colony morphology, color and size. Amongst the 1017 isolates selected for analyses, 49.4 % was derived from the roots, 29.8 % from stems and 20.8 % from leaves. 16S rRNA gene analysis assigned 34.4 % of them to *α*-*Proteobacteria*, 31.1 % *γ*-*Proteobacteria* and 24.5 % *Actinobacteria* (Additional file 3: Table S1; Additional file 4: Table S2).

**Fig. 1:**
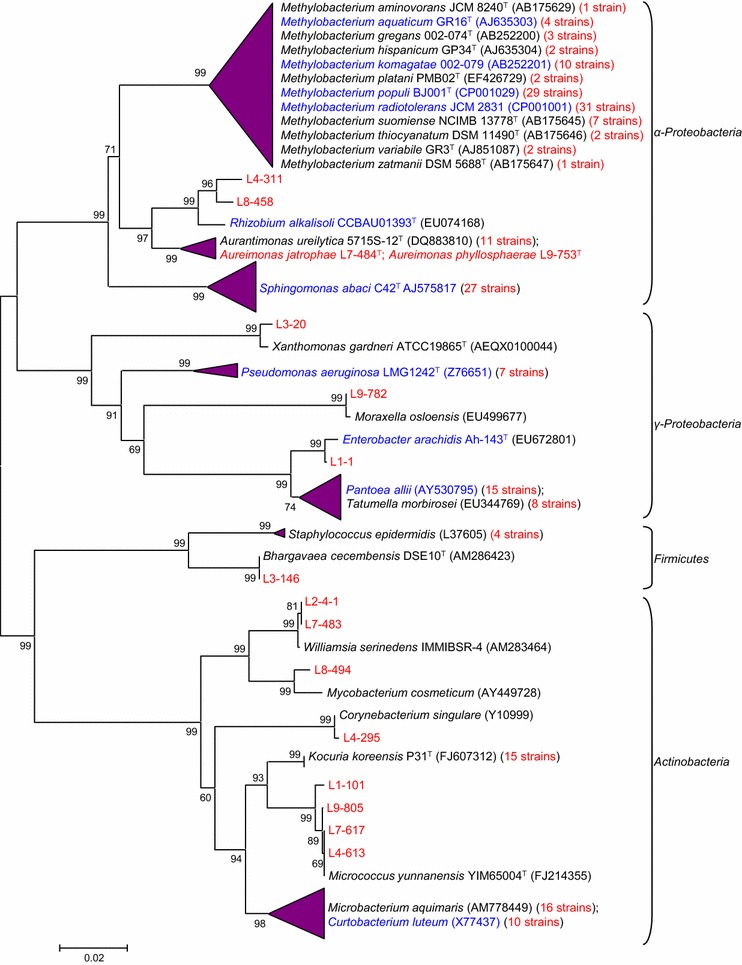
Phylogenetic positions and diversity of leaf-endophytic species. The tree was constructed based on 16S rDNA sequences using the Neighbor-Joining method. Bootstrap values (using 1000 replicates) are indicated at the branching points. *Scale bar* represents % estimated substitutions. Candidates for nitrogen-fixers (shown in *blue*) indicate the presence of *nifH* gene as evidenced by PCR amplifications. The number of strains is shown in *parenthesis in red*. The *arrowhead* sizes indicate the relative abundance of the genus

In leaves, *α*-*Proteobacteria*, particularly *Methylobacterium* genus clearly stood out in the population, while *Sphingomonas, Pantoea, Kocuria, Microbacterium* and *Curtobacterium* genera were also frequently isolated (Fig. 1; Additional file 4: Table S2). The stem population resembled that of leaves, with *Curtobacterium, Methylobacterium* and *Sphingomonas* being the top 3 genera (Additional file 5: Figure S3). In contrast, *Pseudomonadaceae*, *Enterobacteriaceae* and *Rhizobiaceae* dominated in roots (Additional file 6: Figure S4). The growth promoting role of *Enterobacter* in Jatropha has been demonstrated previously [32]. Surprisingly, 31.2 % of the isolates potentially represent new taxa (Additional file 7: Figure S5).

Cell wall degrading endoglucanase activity was believed to be critical for endophytes to successfully colonize plants [35]. We found that 253 strains (24.9 %) exhibited clear zones on CMC plates stained with Congo red, indicating production of endoglucanase in those strains (Table 1, Additional file 3: Table S1). Isolates from genera *Cellulosimicrobium*, *Curtobacterium*, *Kosakonia* and *Pseudomonas* were most frequently observed to produce endoglucanase. Unexpectedly, 55.9 % of the endophytic isolates did not show obvious endoglucanase activity. As it has been suggested previously, many of endophytes may passively entered the system from the root and spread to aerial parts in a systematic manner [36, 37].

**Table 1:** Distribution of nitrogen-fixing and endoglucanase-positive strains originating from different media

Media	Medium	Total number of isolates^a^	Positive isolates (%)		
			*nifH* ^b^	ARA^c^	Endoglucanase^d^
Rich media	M869	257	132 (51.4)	81 (29.5)	98 (38.1)
	KB	354	206 (58.2)	143 (40.4)	155 (43.8)
Heterotrophic media	R2A	189	112 (59.3)	58 (30.7)	75 (39.7)
Minimal media	AMS	106	80 (75.5)	49 (46.2)	33 (31.1)
N-free media	Nfb	101	68 (67.3)	45 (45.6)	78 (77.2)
	Baz	10	5 (50.0)	5 (50.0)	9 (90.0)

^a^Total number of isolates selected for 16S rRNA gene sequencing and phylogeny

^b^PCR amplification of *nifH* gene fragments was performed using specific degenerate primers

^c^Acetylene reduction activity in pure culture was measured by GC

^d^Plate assays for endoglucanase activity using KM solid medium with 0.2 % CMC were spot inoculated with endophytes and incubated at 30 °C for 3 days

### Endophytic nitrogen-fixing bacteria

Among the 1017 isolates, 111 strains (11 %) were able to grow in nitrogen-free media. Members of the genus *Pseudomonas* (35.1 %)*, Curtobacterium* (10.8 %), *Methylobacterium* (9.0 %)*, Sphingomonas* (8.1 %) and *Rhizobium* (8.1 %) were the major taxa overall (Additional file 4: Table S2, Additional file 6: Table S3). To further confirm the diazotrophic nature of the strains, we analyzed the *nifH* gene encoding the dinitrogenase reductase subunit. *nifH* sequences were detected by PCR in 64.8 % of the strains that were able to grow in N-free medium [38]. Notably, only 37.4 % *nifH*-positive strains displayed nitrogenase activity in vitro (Table 2; Additional file 3: Table S1). The discrepancy may be attributed to the presence of non-functional *nifH* gene. Alternatively, nitrogenases were not functional under the in vitro assay conditions. Therefore, the ability to grow on N-free medium and the presence of a *nifH* gene does not warrant nitrogenase activity in vitro. This is in accordance with several previous studies on nitrogen-fixing bacteria [39–41]. The majority of the *nifH*-positive isolates that failed to show in vitro nitrogenase activity belonged to the order *Rhizobiales*: e.g., *Rhizobium*, *Ensifer*, *Sinorhizobium*, *Bradyrhizobium*, and *Mesorhizobium* genera, which are known to fix nitrogen effectively only in root nodules [42, 43]. In contrast, isolates belonging to the genus *Cellulomonas, Curtobacterium, Microbacterium, Mycobacterium, Chryseobacterium* or *Achromobacter* showed AR activity. However, no *nifH* DNA sequences could be amplified under the conditions used, suggesting the *nifH* genes in those isolates was more divergent.

**Table 2:** Distribution of *nifH*-positive representative bacterial taxa in the leaf, stem and root of Jatropha germplasm accessions

Phylogenetic group	Genera (21)	Number of isolates					
		AR-activity positive^a^			AR-activity negative^b^		
		Leaf	Stem	Root	Leaf	Stem	Root
*Actinobacteria*	*Cellulomonas*		1				
	*Curtobacterium*	10	35	1			
	*Microbacterium*				16	21	4
	*Micromonospora*		1	2			
	*Mycobacterium*					1	5
*Bacteroidetes*	*Chryseobacterium*		1	1			
*Firmicutes*	*Paenibacillus*			3			
*α*-*Proteobacteria*	*Bradyrhizobium*						1
	*Ensifer*			1			13
	*Herbaspirillum*			1			
	*Mesorhizobium*					3	
	*Methylobacterium*	63	21	2	30	7	
	*Pleomorphomonas*			1			
	*Rhizobium*			2	2	14	52
	*Sphingomonas*	15	16		12	16	4
*β*-*Proteobacteria*	*Achromobacter*						11
	*Burkholderia*			4			1
*γ*-*Proteobacteria*	*Klebsiella*			1			
	*Kosakonia*	1		8		1	
	*Enterobacter*		1	8			4
	*Pantoea*	4	12	33	11	2	
	*Pseudomonas*	6	16	104	1	8	39
	*Stenotrophomonas*			5			

^a^Number of positive isolates showing ethylene peak was measured by GC

^b^Number of isolates failed to produce ethylene peak or undetectable quantity

Our results demonstrated that Jatropha tissues are associated with an abundant and diverse population of diazotrophs. The differential pattern of diazotrophic population in different parts of Jatropha shared high similarity to those of soybean and potato [42, 44], but it was significantly different from that of switchgrass and wild rice [45].

### Analyses of *nifH* genes

**Fig. 2:**
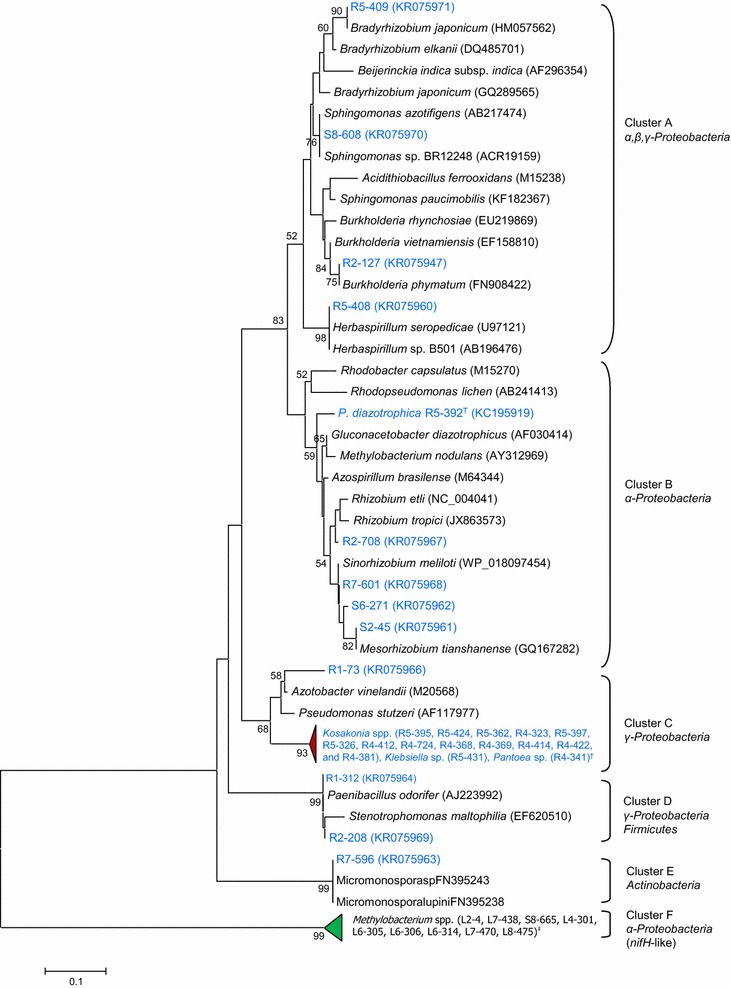
Phylogenetic tree of partial NifH sequences. Alignment was made for the 192 amino acid residues corresponding to amino acid 34–184 in *Azotobacter vinelandii* NifH protein. The tree was constructed using the Neighbor-Joining method. The *scale bar* denotes 0.05 % of sequence distance. The retrieved sequences, in *bold*, were grouped into clusters A, B, C, D, E and F. The *arrowhead* sizes indicate the relative abundance of the genera. Bootstrap values above 50 % are indicated at the branching nodes. Jatropha isolates shown in *light blue color* indicate the presence of *nifH* gene as evidenced by PCR amplifications. The position of root isolates and leaf isolates is marked with *red* and *green arrowheads,* respectively

We sequenced the *nifH* PCR products from 42 strains that appeared to be unique species based on 16S rDNA sequences. All isolates showed AR activity in vitro culture except *Rhizobium* and *Sinorhizobium* groups. Alignment of the predicted NifH amino acid sequences formed 6 major clusters (A–F) (Fig. 2). It is noticeable that the NifH sequence homology does not consistently correlate with phylogenetic relationship. This suggests that multiple independent horizontal gene transfer events occurred in the evolution of nitrogen-fixing bacteria. Notably, cluster F include sequences from leaf isolates only, all belonging to the genus *Methylobacterium*. These sequences were highly divergent from NifH consensus sequence [46]. In fact, they were more related to the Pfam NifH/frxC-family protein, i.e., chlorophyllide reductase iron protein subunit X involved in photosynthesis [47].

### Nitrogenase activity of *Methylobacterium* species in vitro and in planta

**Fig. 3:**
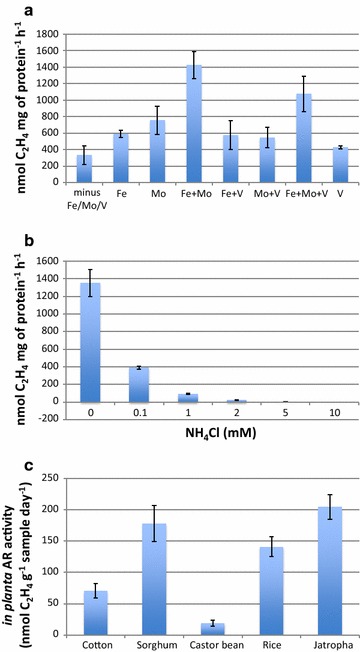
Characterization of nitrogenase. **a**, **b** Acetylene reduction activity in vitro. *Methylobacterium* L2-4 was cultured in N-free medium containing various combination of co-factors (Fe/Mo/V) or concentrations of NH_4_Cl. **a** Effects of co-factors. **b** Sensitivity to ammonium ions. **c** Nitrogenase activity of in planta. Seedlings of various crops were inoculated with L2-4 strain and AR assays were done 20 days after inoculation. *Each value* represents mean ± standard deviation (SD) of three replicates

Among 125 *Methylobacterium* isolates (Additional file 4: Table S2) characterized from Jatropha plant tissues, obvious AR activity was observed in 34 strains (20–634 nmol C_2_H_4_/bottle). 52 strains showed weak AR activity (<20 nmol C_2_H_4_/bottle) while 39 strains had no detectable activity although all strains had the *nifH*-like sequences (Additional file 3: Table S1, Additional file 4: Table S2). Phylogenetically, AR-positive strains were closely related to *M. radiotolerans*, *M. populi*, *M. komagatae* and *M. aquaticum* (Additional file 3: Table S1). Strain L2-4 can be classified as *M. radiotolerans* based on its rDNA sequence and was among the fastest grower in N-limiting conditions and showed distinct AR activity in vitro (Additional file 9: Figure S6). Assays of its AR activity in the presence or absence of iron (Fe^2+^), molybdenum (MoO_4_^2−^) and vanadium (V) suggest that the L2-4 strain nitrogenase used Fe^2+^ and MoO_4_^2−^) as co-factors. The highest AR activity was recorded in the presence of FeSO_4_ (10 mg/l) and Na_2_MoO_4_ (5 mg/l) (Fig. 3a). Vanadium showed weak inhibitory effect. As expected, ammonium ion strongly inhibited AR activity (Fig. 3b). The ability of L2-4 strain to fix nitrogen in planta was confirmed by inoculating the strain to Jatropha by foliar spraying and maintaining the plants under sterile condition. Seedlings treated with strain L2-4 showed strong AR activity (204.6 nmol C_2_H_4_ g^−1^ dry tissues day^−1^). Furthermore, L2-4 strain also displayed strong AR activity in planta in sorghum, rice, cotton, and caster plant (Fig. 3c). The association between *Methylobacterium* species and host plants varies from strong or symbiotic to weak or epiphytic and to intermediate or endophytic [48, 49]. *Methylobacterium nodulans* and *M.radiotolerans* have been reported to be involved in nitrogen fixation and nodule formation [50, 51], while other *Methylobacterium* species has been reported multiple plant growth promoting traits [52, 53].

### Epiphytic and endophytic colonization by *Methylobacterium*

*Methylobacterium* species have been found in association with several species of plants, actively colonizing leaves, stem, branches and roots [54–60]*. Methylobacterium* cells were observed in intracellular space of the meristematic cells of Scots pine and tomato [61, 62]. Endophytic occurrence of *Methylobacterium* was confirmed in *Medicago truncatula* leaves [63]. We found that strain L2-4 colonized on leaf surfaces (epiphytic) as well as inside leaf tissue of Jatropha grown under sterile conditions. On day 45 after leaf spraying, surface-sterilized leaf tissues had endophytic bacteria counts of 5.2 × 10^6^ cfu/g leaf tissues. Epiphytic population based on leaf-imprinting assay was >100 cfu (cm^2^)^−1^ in treated leaves. As expected, pink color colonies were not detected in non-treated leaves of Jatropha seedlings grown under sterile conditions.

### Inoculation of *Methylobacterium* improved production of biomass and seeds

**Fig. 4:**
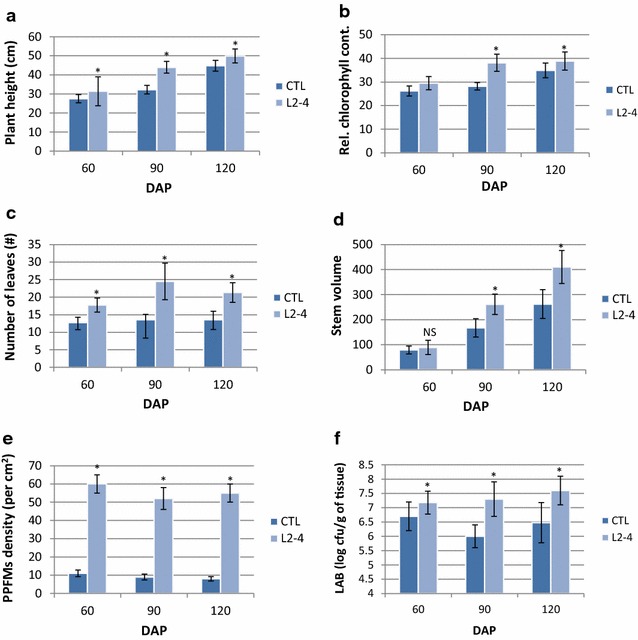
Promotion of Jatropha biomass growth. Jatropha seedlings were inoculated by leaf spraying, planted in large pots and maintained in the open air. Sterilized and non-sterilized garden soil was used in Trail I and II, respectively. Values are mean ± standard deviation (SD). **a** Plant height; **b** relative chlorophyll content; **c** number of leaves; **d** stem volume; **e** pink-pigmented facultative methylotrophic bacteria population; **f** leaf-associated bacteria. *Asterisk* means significant difference at 5 % threshold between treated and control using DMRT. *NS* not significant

**Fig. 5:**
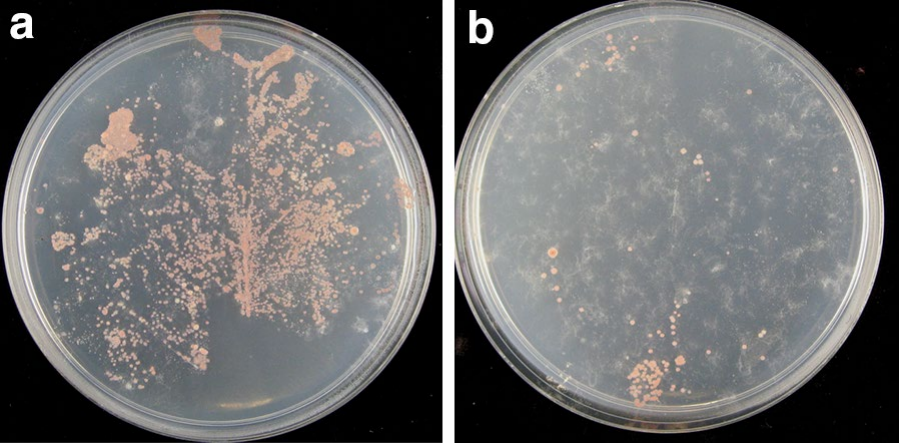
Leaf imprinting. The systemic leaves of L2-4 inoculated plants (at 30 DAI) were printed on an ammonium mineral salts plate supplemented with 0.5 % methanol (v/v) and incubated at 30 °C for 4 days. **a**, **b** show a leaf from an inoculated plant and control plant, respectively

To further confirm the growth promoting effect of strain L2-4, Jatropha seedlings were inoculated with the bacterial suspension through seed soaking and as foliage spray. Seed treatment by soaking seeds with L2-4 bacterial suspension for 2 h increased germination rate by 24 %, from 49.6 % in mock-inoculated seeds to 61.5 % in treated seeds. At 45 days after sowing, the average dry biomass of inoculated plants was 40.1 % higher than the mock-inoculated plants and this was associated with significantly increased leaf chlorophyll content and seedling vigor (Table 3). Several studies reported that *Methylobacterium* inoculation through seed imbibition and phyllosphere spray enhanced seed germination rate, storability, and seed vigor [64–66]. In another word, *Methylobacterium* has both nurturing and protecting roles for the plants [67]. To demonstrate that nitrogen-fixing strain L2-4 is able to improve seed production of Jatropha, seedlings were inoculated by leaf spraying, planted in large pots and maintained in the open air. Again, L2-4 treated plants showed significant improvements in plant height, leaf counts, leaf chlorophyll content and stem volume compared with the untreated control plants (Fig. 4). At 120 DAI, treated plants recorded an increase of 11.5, 57.1, 11.4 and 56.2 % over the mock-inoculated controls in plant height, leaf counts, leaf chlorophyll content and stem volume, respectively (Fig. 4a–d). In consistence with the plant growth promotion, leaf-epiphytic and endophytic populations were found significantly higher in inoculated plants. Total leaf-associated methylotrophic bacterial density ranged from 7 to 7.5 log cfu g^−1^ of tissues in treated leaves compared to 6–6.7 log cfu g^−1^ of tissues in mock-treated plants at 60–120 DAP (Fig. 4e, f). Leaf-imprinting confirmed that strain L2-4 was an epiphyte, displaying 50–60 cfu (cm^2^)^−1^ in treated leaves compared to 8–11 cfu (cm^2^)^−1^ in non-treated leaves (Fig. 5). We analyzed the 16S rRNA sequence of 20 randomly picked colonies from the leaf-imprinting (Fig. 5a) and 15 of them (75 %) were identical to that of L2-4. These results indicate that L2-4 strain competed well with indigenous phyllosphere microflora under non-sterile conditions. As expected, pink-pigmented *Methylobacterium* were detected in low density in roots or stems irrespective of L2-4 treatments (data not shown).

**Table 3:** Effects of L2-4 strain inoculation on the early growth parameters of Jatropha

Treatments^a^	SVI^b^	Relative chlorophyll content	Seedling dry biomass (per seedling)^c^
Control	1818.4 ± 69.3	34.91 ± 1.79	3.07 ± 0.27
L2-4	3297.7 ± 153.7	38.81 ± 0.51	4.30 ± 0.18
LSD (*P* ≤ 0.05)	551.02	3.18	0.32

^a^After seed soaking, the seeds (50 seeds/replicate, n = 3) were drained and sown in trays containing non-sterilized soil and maintained in a greenhouse and at 28 °C

^b^Seedling vigor index (SVI) was calculated using the formula: SVI = % germination × seedling length (shoot length + root length) in cm

^c^Each value represents mean of three replicates and expressed in grams. Samples were measured at 45 DAS

In two independent long-term open-air growth experiments using sterilized and non-sterilized soil, the average seed set per tree was increased by approximately 213 and 84.3 %, respectively (Table 4). Student’s *t* test showed that the treated plants produced significantly more seed sets than mock-treated ones in both experiments (*P* < 0.05). The improvement in seed yield was associated with an increase of female-male flower ratio and fruit sets (Table 4). The average single seed weight was increased by 12.2 % in Trial I and 11.3 % in Trial II, both being very significant according to Student’s *t* test (*P* < 0.01). Cytokinin has been shown to improve female-to-male flower ratio [15] and *Methylobacterium* has been shown to change auxin and ethylene levels in plants due to secretion of 1-aminocyclopropane-1-carboxylate (ACC) deaminase [68]. The cross-talk between cytokinin and ethylene pathways is well established. *Methylobacterium* strain L2-4 appears to promote Jatropha growth and seed setting via multiple mechanisms, including nitrogen fixation, modulating photosynthesis, leaf senescence and flower sex differentiation. The genome of strain L2-4 presents several genes involved in metabolic pathways that may contribute to promotion of plant growth and adaptation to plant surfaces [46]. *Methylobacterium* on plant surfaces benefit from methanol produced by plants by means of methylotrophy [59, 69, 70]. However, methanol is not the only carbon substrate that these bacteria are able to consume in the phyllosphere [63].

**Table 4:** Effects of L2-4 strain inoculation on flower sex ratio and seed yield parameters of Jatropha

Treatment	Number of female flowers per inflorescence^a^	Number of male flowers per inflorescence^a^	Ratio female:male flower	Number of fruits^b^	Number of seeds^b^	Seed weight (g)^b^	per seed weight (mg)*
Trail I							
Control	1.68 ± 0.14	25.2 ± 1.83	1:15	4.75 ± 0.71	13.5 ± 2.13	6.86 ± 0.91	426.5 ± 41.8
L2-4	4.48 ± 0.62	39.2 ± 4.19	1:9	16.6 ± 1.19	42.3 ± 4.27	22.1 ± 2.04	478.7 ± 20.7
LSD (*P* ≤ 0.05)	0.75	4.08		1.13	4.12	1.93	
Trail II							
Control	3.5 ± 0.07	55.0 ± 2.08	1:16	10.7 ± 1.15	28.6 ± 3.78	13.6 ± 0.51	424.1 ± 25.2
L2-4	9.28 ± 0.19	72.3 ± 1.99	1:8	18.4 ± 1.98	52.7 ± 4.72	26.7 ± 0.84	472.0 ± 13.0
LSD (*P* ≤ 0.05)	1.92	8.18		1.50	3.57	2.39	

Seedlings were inoculated by foliar spraying at 21 days after seed germination. A second spraying was made at the flowering stage. Plants were planted in large pots (n = 8 in Trial I and n = 12 in Trial II) and maintained in the open air

Values are mean ± standard deviation (SD). Sterilized and non-sterilized garden soil was used in Trail I and II, respectively

^a^Data were recorded with 25 and 50 inflorescences at different time points for Trail I and II, respectively

^b^Number of fruits/plant, number of seeds/plant and seed weight/plant were recorded on 480 and 540 DAI from Trail I and II, respectively

* Student’s *t* test showed that trees treated with L2-4 had significantly higher seed sets and seed yield per plant than non-treated controls (*P* < 0.05)

## Conclusions

We have provided strong evidence that the dominant leaf-associated *Methylobacterium* species were able to promote Jatropha growth and seed yield, at least in part due to nitrogen fixation. To the best of our knowledge, this is the first report of bacterial nitrogen fixation on leaf surface although strain L2-4 is also a competent endophyte in Jatropha. The abundance of endophytic nitrogen-fixing bacteria in Jatropha may contribute to Jatropha’s strong tolerance to poor soil nutrient. Our studies also suggest that strain L2-4 is able to promote growth and perform nitrogen fixation in a much wider range of crops.

## Methods

### Sampling and isolation of endophytic bacteria

Jatropha germplasm accessions collected in the form of seeds from Maluku Island, Indonesia; Yunnan Province, China; and Madurai, Tamil Nadu, India, and the plants were maintained at the Agrotechnology Experimental Station, Singapore. These natural germplasms accessions have been selected in the breeding program of JOil Company (http://www.joil.com.sg/) to generate hybrid plants on the basis of high productivity [71]. Healthy, symptom-less leaves, stems and roots were collected from three individual plants of each germplasm and treated separately. Lateral roots of approximately 15 cm away from the primary stems with diameters from 0.5 to 1.5 cm were collected. Fully expanded leaves with no obvious pathogenic infections were collected. Similarly, uninfected stem segments of about 2.0–2.5 cm in diameter were sampled. All tissues were washed with 70 % ethanol at the cut sites and placed in plastic bags on ice during transportation. Subsequently, samples were subjected to a two-step surface sterilization procedure by washing for 5 min in 1 % (w/v) sodium hypochlorite supplemented with 1 drop of Tween 80 per 100 ml solution followed by three rinses in 70 % ethanol in sterilized distilled for 1 min each. To ensure complete surface sterilization, a second treatment was performed by washing the tissues for 15 min in 15 % H_2_O_2_, followed by 1 min in 70 % ethanol, and then rinsed in sterilized distilled water. A 100 µl sample of the water from the third rinse was plated on rich medium to verify the efficiency of sterilization. Surface-sterilized tissues were macerated by grinding in 50 ml 10 mM MgSO_4_ and serially diluted suspensions were plated on various solid media with 15 g/l agar or phytagel, including 869 medium [33], R2A medium [72], King’s B medium [73] and Ammonium Mineral Salt (AMS) medium [74], Nfb medium [75] and BAz medium [76]. Nitrogen-fixing bacterial populations were estimated by the Most Probable Number (MPN) technique using five tubes per dilution with duplicate tubes per dilution [77], and incubated at 30 °C for 4–5 days. Bacterial growth as seen by a fine subsurface pellicle in the tubes were further purified by transferring to an N-free semi-solid medium, and single colonies were isolated by streaking on respective N-free agar plates. For N-free semi-solid medium, MPN counts were calculated at a level of 95 % confidence according to the method previously described [78].

## 16S rRNA gene amplification, sequencing, and strain identification

Phylogenetic positions of bacterial isolates were determined by sequence analysis of the complete 16S rRNA genes. Genomic DNAs were prepared as described previously [79]. 16S rRNA genes were amplified by PCR using universal primers 27F and 1492R [80] (all primer sequences are shown in Additional file 10: Table S4) with the following cycling conditions: initial denaturation for 10 min at 95 °C; 30 cycles of 1.5 min at 95 °C, 1.5 min at 55 °C and 1.5 min at 72 °C; and a final extension for 10 min at 72 °C. PCR products were gel-purified and sequenced directly or cloned in pGEM-T Easy (Promega, Madison, USA) before sequencing with the Big-dye sequencing method (AB Applied Biosystems, Hitachi) using primers 27F, 1492R, 785F, 518R and 1100R. Sequences were aligned with the Megalign program of DNASTAR and analyzed against the EzTaxon-e Database (http://www.ezbiocloud.net/eztaxon) [81]. Phylogenetic analyses were performed by the Neighbor-Joining [82], Maximum-Likelihood [83] and Maximum-Parsimony [84] methods using the MEGA version 5.05 [85] with the bootstrap values set at 1000 replications [83].

### Screening for cell wall degrading endoglucanase activity

Endoglucanase activity was determined as described previously [86] with some modifications. Plates containing Kim-Wimpenny solid medium with 0.2 % carboxymethyl cellulose (CMC) [87], with or without 0.5 % d-glucose, were spotted with 1 µl of grown cultures (OD_600nm_ = 1.0), air-dried and incubated at 30 °C for 3 days. Cell colonies were flushed off plates with water and plates were stained with a 0.1 % Congo red solution for 30 min, followed by several washes with 1 M NaCl. The appearance of clear yellow halo around the colony in a red background indicates positive staining for endoglucanase activity.

### Nitrogenase activity assay and *nifH* gene screening

Nitrogen-fixing capability of isolated strains was screened by testing their growth in 2 ml nitrogen-free liquid medium as described previously [32]. Nitrogenase activity of selected strains was confirmed by acetylene reduction assay (ARA) in liquid cultures injected with purified acetylene gas (15  % v/v) in gas-tight bottles, which were incubated up to 96 h at 30 °C. Gas samples (0.5 ml) were extracted at regular intervals with a PTFE-syringe (Hewlett-Packard, USA) and analyzed in a Gas Chromatograph (GC 6890 N, Agilent Technologies Inc., USA) with an FID operated under the following conditions: carrier gas: He-35 ml/min; detector temperature: 200 °C; column: GS-Alumina (30 m × 0.53 mm I.D.); pressure: 4.0psi. Ethylene produced by the bacteria was quantified using standard ethylene (C_2_H_4_, Product Number: 00489, Sigma-Aldrich) curve prepared in duplicates in concentrations ranging from 1 to 1000 nmol. Protein concentration was determined with a modified Lowry method using BSA as the standard. For nitrogenase switch-off/switch-on assay, *Methylobacterium* cells were grown in N-free medium containing different levels (0–10 mM) of ammonium chloride and nitrogenase co-factors FeSO_4_ (10 mg l^−1^), Na_2_MoO_4_ (5 mg l^−1^) and VCl_2_ (18.1 mg l^−1^). Acetylene reduction activity in planta was performed as described previously [32]. Briefly, samples from each replication were collected from the glass house and most of the adhering soil was removed by shaking. Seedlings were inserted into the 125 ml glass bottles, closed with a 20 mm red stopper sleeve. After removing an equivalent volume of air, acetylene was injected into these bottles to give a final concentration of 15 % and incubated at 30 °C for 24 h. In planta acetylene reduction activity was measured by GC and value is expressed in nmol C_2_H_4_ released day^−1^ seedlings^−1^ after subtracting plant’s background C_2_H_4_ emission.

PCR amplification of *nifH* gene fragments was performed using primers nif-Fo and nif-Re under stringent cycling conditions as described [88], i.e., 95 °C/5 min, 40 cycles of 94 °C/11 s, 92 °C/15 s, 54 °C/8 s, 56 °C/30 s, 74 °C/10 s and 72 °C/10 s, and final extension for 10 min/72 °C. PCR products were purified with QIAquick gel extraction kit (Qiagen, USA) and sequenced.

### Leaf colonization by *Methylobacterium*

Surface sterilization of Jatropha seeds was done by washing coat-less seed kernels in 90 % ethanol (v/v) for 1 min and 10 % H_2_O_2_ (v/v) for 60 min followed by 3-5 rinses in sterilized distilled water. After soaking overnight at 28 °C in darkness, they were germinated on a hormone-free seed germination medium [32] in Petri dishes and incubated at 25 °C with 16/8 h light–dark cycles. After 10 days, healthy seedlings (10 seedlings/replica, n = 3) were transferred to Phytatrays (Sigma, USA) containing sterile sand (autoclaved) with 40 ml of plant nutrient solution [89]. Jatropha leaves were sprayed with L2-4 suspension (10^8^ cfu/ml) till completely wet. On day 45 and 60, epiphytic population was determined from leaves of inoculated plants were printed on an AMS agar plate supplemented with 0.5 % methanol (v/v) and incubated at 30 °C for 3–5 days. Endophytic colonization was determined from surface-sterilized leaves and homogenize with sterile pestle and mortar followed by serial dilution methods. Serially diluted samples were plated on an AMS agar plates, incubate at 30 °C for 3–5 days and pink-pigmented colonies counted from 10^−3^ to 10^−4^ dilutions.

### Effect of *Methylobacterium* L2-4 on Jatropha seedling early growth

Seedling vigor test was performed with Jatropha to study effects of foliar spray with ACC deaminase producing *Methylobacterium*. Strain L2-4 was cultured in 2YT broth supplemented with 1 % methanol (v/v) until exponential growth phase and harvested by centrifugation. After washing once with sterile distilled water, inoculants were made by re-suspending the pellets in water to an OD_600nm_ of 1.2 (~10^8^ cfu ml^−1^). To assess the impact on seed germination and early growth of seedlings, imbibed seeds (50 seeds/replica, *n* = 3) were sown in plastic pots individually and allowed to develop into seedlings. Foliar application was done after seed germination and growth parameters were recorded at 45 DAS.

### Effect of *Methylobacterium* on Jatropha growth and seed yield

Seeds of *J. curcas* cv. MD44 were used throughout the experiments. Surface sterilization of seeds was done by washing coat-less seed kernels in 75 % ethanol (v/v) for 1 min and 10 % H_2_O_2_ (v/v) for 60 min followed by 3–5 rinses in sterilized distilled water. After soaking overnight at 28 °C in darkness, they were germinated on a hormone-free seed germination medium (1/2 MS salt, B5 vitamins, 5 g l^−1^ sucrose, 0.5 g l^−1^ MES and 2.2 g l^−1^ phytagel, pH 5.6) in Phytatrays (Sigma, USA) in a tissue culture room with a temperature of 25 ± 2 °C and 16/8 h light–dark cycles. To assess the effects of bacterial inoculation on the growth and yield of Jatropha under natural conditions, two pot culture experiments were conducted with garden soil. Plants were planted in pots (one plant per pot) in sterilized soil (compost/sand mix at 1:1 ratio and in ɸ23 cm, 18 cm height pots; named as Trial I) or non-sterilized soil (nutrient poor clay soil in ɸ30 cm, 28 cm height pots; named as Trial II). Trial I and Trial II were maintained in different locations and started in different seasons. L2-4 cell suspension (1.2 OD_600_) was applied as foliar spray till wetting of the leaves at 21 days after seed germination. Commercial NPK Fertiliser was applied once in 15 days at about half of the recommended dose of approximately 50:30:30 g^−1^ plant^−1^ year^−1^. Biometric observations were recorded once in 30 days. After flowering, yield parameters were recorded once in 30 days. Seed set numbers per plant (*n* = 9 in Trial I and *n* = 12 in Trial II) were measured at 480 and 520 DAI in Trail I and Trail II, respectively, and single seed weight was calculated based on the average of 180 randomly selected seeds per treatment were measured.

Triplicate leaf samples were randomly picked from three plants on 30 DAI. For methylotrophic bacterial enumerations, homogenates were serially diluted using 1X PBS and plated on to AMS media with 0.5 % methanol to determine the methylotrophic population. Pink-pigmented colonies were counted after incubating the plates for 5 days at 30 °C. Further confirmation, 20 pink color colonies per replica were picked from 10^−5^ dilution and streaked on AMS agar plates and purified. Purified colonies were sequenced by 16S rRNA sequencing and identified using the EzTaxon server [81] on the basis of sequence data and sequencing results compared with pairwise identity of strain L2-4.

### Statistical analysis

Statistical analyses were carried out using the Statistical Analysis System (SAS) Version 9.2 (SAS Institute Inc., Cary, North Carolina, USA). Analysis of variance (ANOVA) for the endophytic and total bacterial population was carried out using the General Linear Model, GLM in SAS. The bacterial population data were log transformed before being subjected to further analysis. The means of the treatment results were subjected to ANOVA and presented using Fisher’s protected Least Significant Difference (LSD). The model adopted was A [log CFU (g/FW)] = C (cultivar) Pt (plant tissue) M (medium) C*Pt C*M Pt*M to check the effect of individual factors and the interactions between them. A canonical discriminant analysis was carried out to discriminate the variations among the cultivars or plant tissue with reference to the endophytic and total population. Given two or more groups of observation with measurements on several quantitative variables, CDA derives a linear combination of the variables that have the highest possible multiple correlation with the groups. Endophytic bacterial inoculation data were subjected to analysis of variance and testing of means by Duncan’s Multiple Range Test (DMRT) at *P* ≤ 0.05 using SAS package. Student’s *t* test was done using the JavaScript maintained by Professor Hossein Arsham, Johns Hopkins Carey Business School (http://home.ubalt.edu/ntsbarsh/Business-stat/otherapplets/MeanTest.htm).

### Nucleotide sequence accession numbers

All 16S rRNA gene sequences determined in this study have been submitted to NCBI under the accession numbers JQ659304 to JQ660320 and the numbers are also listed in Additional file 3: Table S1. *nifH* gene sequences have been submitted to NCBI under the accession numbers KR075947-KR075982, KC195919 and CP005991.

## Additional files

10.1186/s13068-015-0404-y Culturable endophytic bacteria densities in various Jatropha tissues. Surface-sterilized tissues (roots, stems and leaves) were grinded into fine powder; diluted in water in series and plated on to different media. Values shown are the average of three individual plants originating from the same germplasm collection. (a) Germplasm from Indonesia, (b) Germplasm from China and (c) Germplasm from India. Each value represents the mean ± SD, n = 3.
10.1186/s13068-015-0404-y Discriminant function analysis. Ordination plots of variables resulting from the first (CAN1) and second (CAN2) canonical functions for different plant tissue types (a) and media (b). The variables were generated based on the total populations from different plant tissues (leaf, stem and root) and media (HTM, NFM and MM).
10.1186/s13068-015-0404-y Complete table of taxonomic units (strains) derived from surface sterilized Jatropha tissues. Table includes, strains, cultivars, plant tissue, growth media, close relatives, pairwise similarity, class, GenBank ID number, nifH positive, endoglucanase and N-fixing activity. *EzBioCloud software to identify closely related type strains (Kim et al., 2012). The media 869, R2A and King’S B medium used for isolation of heterotrophic bacteria; AMS medium used for isolation of methylotrophic bacteria; N-free semisolid used for isolation of diazotrophic bacteria.
10.1186/s13068-015-0404-y Distribution of representative bacterial taxa in the leaf, stem and root of Jatropha biodiesel plants. Relative abundance of different taxon at genus level based on 16S rRNA gene sequences under different classes or phyla.
10.1186/s13068-015-0404-y Phylogenetic positions of stem endophytes. The tree was constructed based on the 16S rDNA sequences using the Neighbor-Joining method. Bootstrap values (using 1000 replicates) are indicated at the branching points. Scale bar indicates  % estimated substitutions. Candidate for nitrogen-fixers (shown in blue) indicate the presence of *nifH* gene as evidenced by PCR amplifications. The number of strains is shown in parenthesis in red. The green arrowhead sizes indicate the relative abundance of the genus.
10.1186/s13068-015-0404-y Phylogenetic positions of root endophytes. The tree was constructed based on the 16S rDNA sequences using the Neighbor-Joining method. Bootstrap values (using 1000 replicates) are indicated at the branching points. Scale bar represents  % estimated substitutions. Candidate for nitrogen-fixers (shown in blue) indicate the presence of *nifH* gene as evidenced by PCR amplifications. The number of strains is shown in parenthesis in red. The yellow arrowhead sizes indicate the relative abundance of the genus.
10.1186/s13068-015-0404-y Cultivable endophytic bacterial diversity of Jatropha. Distribution of the phylotypes (%) over the different phyla and classes. The bar of each phylogenetic group is subdivided according to the different identification levels of the phylotypes. The group “identified at species level” contains those phylotypes that belong to existing species with a 16S rDNA homology threshold of ≥ 99.0 %. The group “identified at genus level” contains phylotypes that, based on phylogeny, belong to a particular genus and may represent a new species within that particular genus or an existing species within this genus showing < 99 % 16S rRNA gene sequence pairwise similarity with the type strain of this species. The group “potential gen. nov.” contains those phylotypes that could not be assigned to a particular genus (< 97 % pairwise similarity) based on the phylogeny of the 16S rRNA gene.
10.1186/s13068-015-0404-y Distribution of representative bacterial taxa in the different germplasm of Jatropha biodiesel plants. Relative abundance of different taxon at genus level based on 16S rRNA gene sequences under different classes or phyla.
10.1186/s13068-015-0404-y Gas chromatography chromatogram showing ethylene and acetylene peaks. (a) 0.5 ml of 4.85 μmol ethylene (C_2_H_4_, Product Number: 00489, Sigma-Aldrich) standard was injected in GC. (b) Strain L2-4 inoculated in N-free medium (40 ml) and ARA was performed by injecting purified acetylene into the bottles sealed with gas-tight serum stoppers to yield 15 % acetylene (v/v); this was followed by incubation for up to 48 h at 30 °C. (c) ARA was performed without strain L2-4 (blank).
10.1186/s13068-015-0404-y List of primers used in this study.

## Abbreviations

ACC: 1-aminocyclopropane-1-carboxylate
AMS: ammonium mineral salt medium
ANOVA: analysis of variance
ARA: acetylene reduction activity
CDA: canonical discriminant analysis
CFU: colony forming units
CMC: carboxymethyl cellulose
DAI: days after inoculation
DMRT: Duncan’s multiple range test
LSD: least significant difference
MPN: most probable number
PGP: plant growth promotion
RG: rate of germination
SAS: statistical analysis system
SVI: seedling vigor index
