# Supplementary material for: Leaf-residing Methylobacterium species fix nitrogen and promote biomass and seed production in Jatropha curcas
Source: Biotechnol Biofuels. 2015 Dec 21;8:222. doi: 10.1186/s13068-015-0404-y (PMC4687150; doi:10.1186/s13068-015-0404-y)
Supplement: Supplementary file 1 — 10.1186/s13068-015-0404-y Culturable endophytic bacteria densities in various Jatropha tissues. Surface-sterilized tissues (roots, stems and leaves) were grinded into fine powder; diluted in water in series and plated on to different media. Values shown are the average of three individual plants originating from the same germplasm collection. (a) Germplasm from Indonesia, (b) Germplasm from China and (c) Germplasm from India. Each value represents the mean ± SD, n = 3. [file 13068_2015_404_MOESM1_ESM.pptx]

## Slide 1
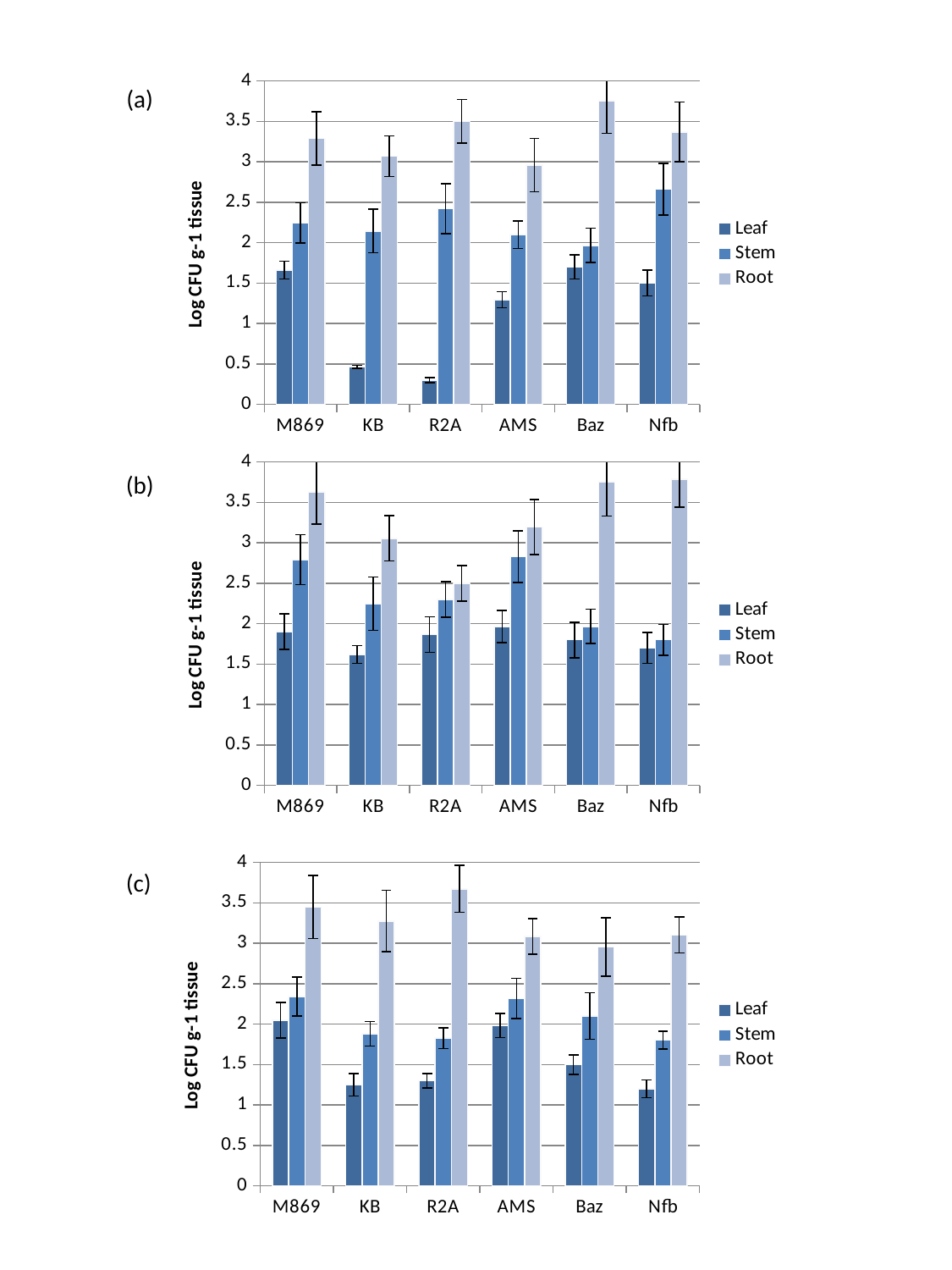

### Chart
| Category | | | |
|---|---|---|---|
| M869 | 1.6600000000000001 | 2.245 | 3.29 |
| KB | 0.465 | 2.145 | 3.07 |
| R2A | 0.3000000000000001 | 2.42 | 3.5 |
| AMS | 1.2949999999999935 | 2.1 | 2.96 |
| Baz | 1.700000000000001 | 1.9675789929999967 | 3.7529588759999997 |
| Nfb | 1.5 | 2.66 | 3.3699999999999997 |(a)
### Chart
| Category | | | |
|---|---|---|---|
| M869 | 1.9000000000000001 | 2.7925454174999977 | 3.6296918750000002 |
| KB | 1.616838333 | 2.246530207500021 | 3.0559139575 |
| R2A | 1.8666666669999998 | 2.3 | 2.4980515309999998 |
| AMS | 1.9664950005000001 | 2.830035207 | 3.1965302075000137 |
| Baz | 1.8 | 1.967578993 | 3.7529588759999997 |
| Nfb | 1.7 | 1.8 | 3.78896945 |(b)
### Chart
| Category | | | |
|---|---|---|---|
| M869 | 2.0466666664999997 | 2.3427170834999997 | 3.446358542 |
| KB | 1.25 | 1.879863541 | 3.275 |
| R2A | 1.3 | 1.825707085 | 3.672344446 |
| AMS | 1.9833333335 | 2.3170099984999997 | 3.0833333335 |
| Baz | 1.5 | 2.1 | 2.952444164999982 |
| Nfb | 1.2 | 1.8 | 3.102776532 |(c)
