# Supplementary figures and images for: Leaf-residing Methylobacterium species fix nitrogen and promote biomass and seed production in Jatropha curcas
Source: Biotechnol Biofuels. 2015 Dec 21;8:222. doi: 10.1186/s13068-015-0404-y (PMC4687150; doi:10.1186/s13068-015-0404-y)

## Slide 1
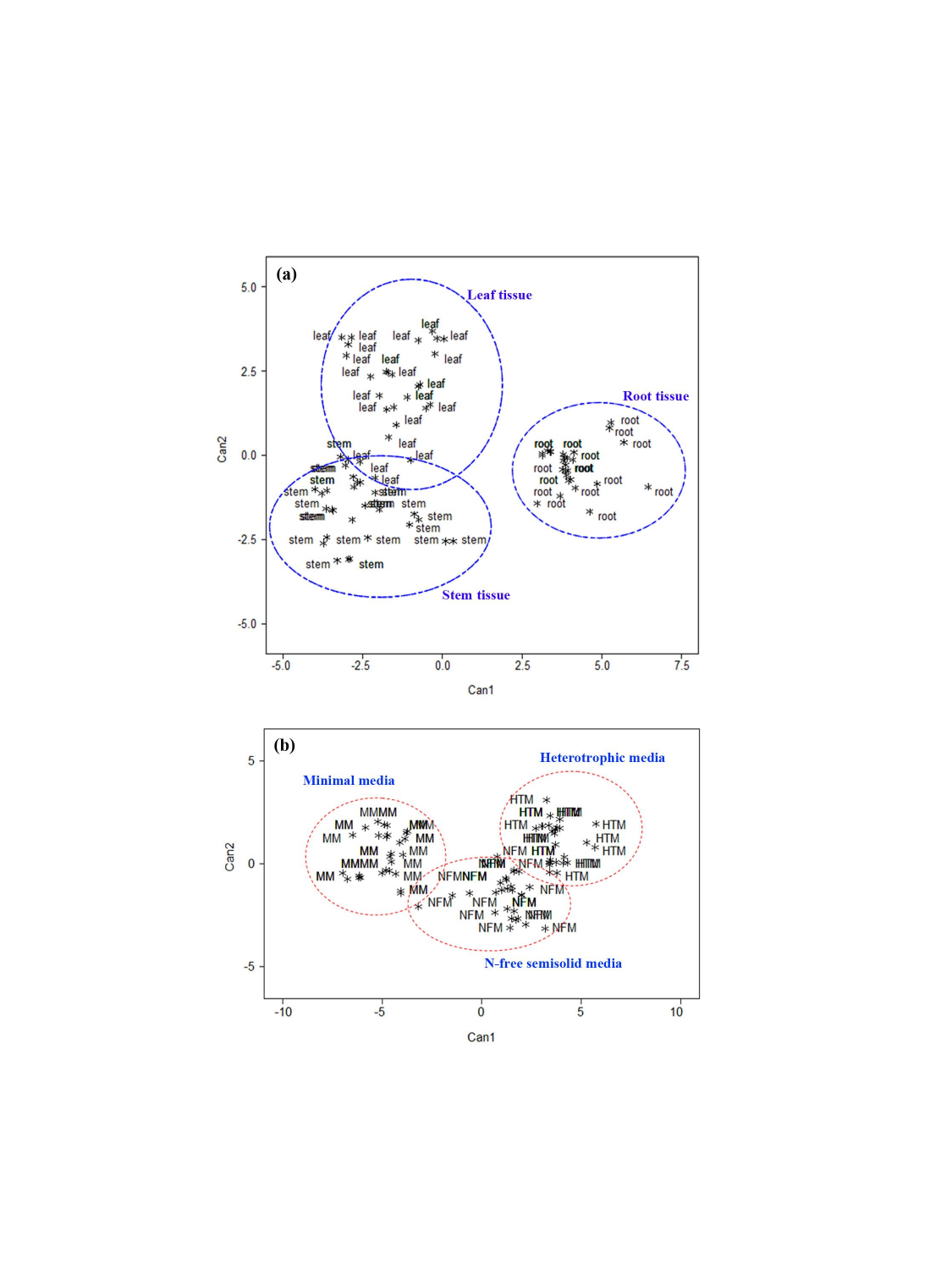

Supplement: Supplementary file 2 — 10.1186/s13068-015-0404-y Discriminant function analysis. Ordination plots of variables resulting from the first (CAN1) and second (CAN2) canonical functions for different plant tissue types (a) and media (b). The variables were generated based on the total populations from different plant tissues (leaf, stem and root) and media (HTM, NFM and MM). [file 13068_2015_404_MOESM2_ESM.pptx]

## Slide 1
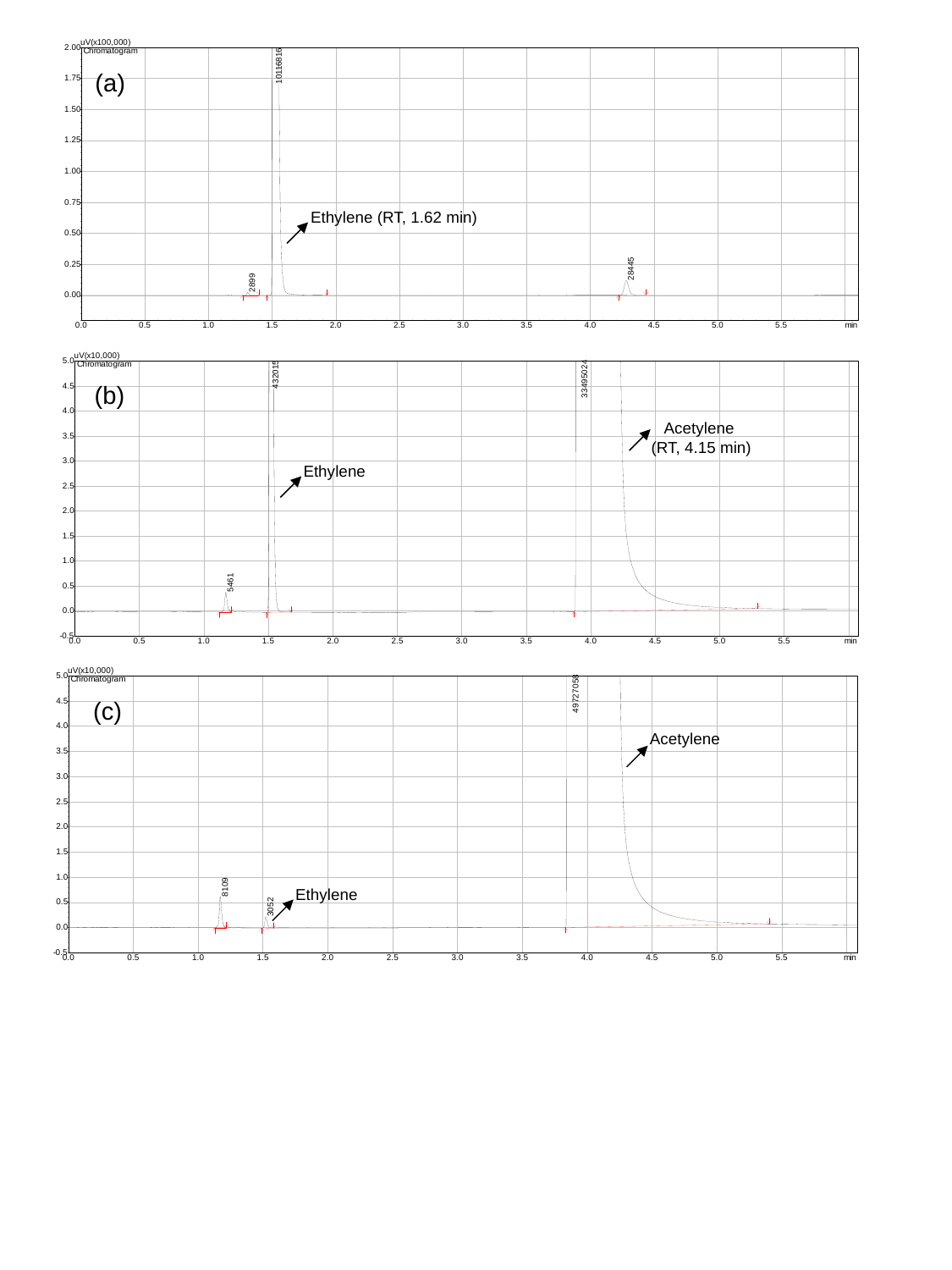

(a)
Ethylene (RT, 1.62 min)
(b)
Acetylene
(RT, 4.15 min)
Ethylene
(c)
Acetylene
Ethylene

Supplement: Supplementary file 9 — 10.1186/s13068-015-0404-y Gas chromatography chromatogram showing ethylene and acetylene peaks. (a) 0.5 ml of 4.85 μmol ethylene (C2H4, Product Number: 00489, Sigma-Aldrich) standard was injected in GC. (b) Strain L2-4 inoculated in N-free medium (40 ml) and ARA was performed by injecting purified acetylene into the bottles sealed with gas-tight serum stoppers to yield 15 % acetylene (v/v); this was followed by incubation for up to 48 h at 30 °C. (c) ARA was performed without strain L2-4 (blank). [file 13068_2015_404_MOESM9_ESM.pptx]
