# Supplementary material for: Leaf-residing Methylobacterium species fix nitrogen and promote biomass and seed production in Jatropha curcas
Source: Biotechnol Biofuels. 2015 Dec 21;8:222. doi: 10.1186/s13068-015-0404-y (PMC4687150; doi:10.1186/s13068-015-0404-y)
Supplement: Supplementary file 5 — 10.1186/s13068-015-0404-y Phylogenetic positions of stem endophytes. The tree was constructed based on the 16S rDNA sequences using the Neighbor-Joining method. Bootstrap values (using 1000 replicates) are indicated at the branching points. Scale bar indicates % estimated substitutions. Candidate for nitrogen-fixers (shown in blue) indicate the presence of nifH gene as evidenced by PCR amplifications. The number of strains is shown in parenthesis in red. The green arrowhead sizes indicate the relative abundance of the genus. [file 13068_2015_404_MOESM5_ESM.pptx]

## Slide 1
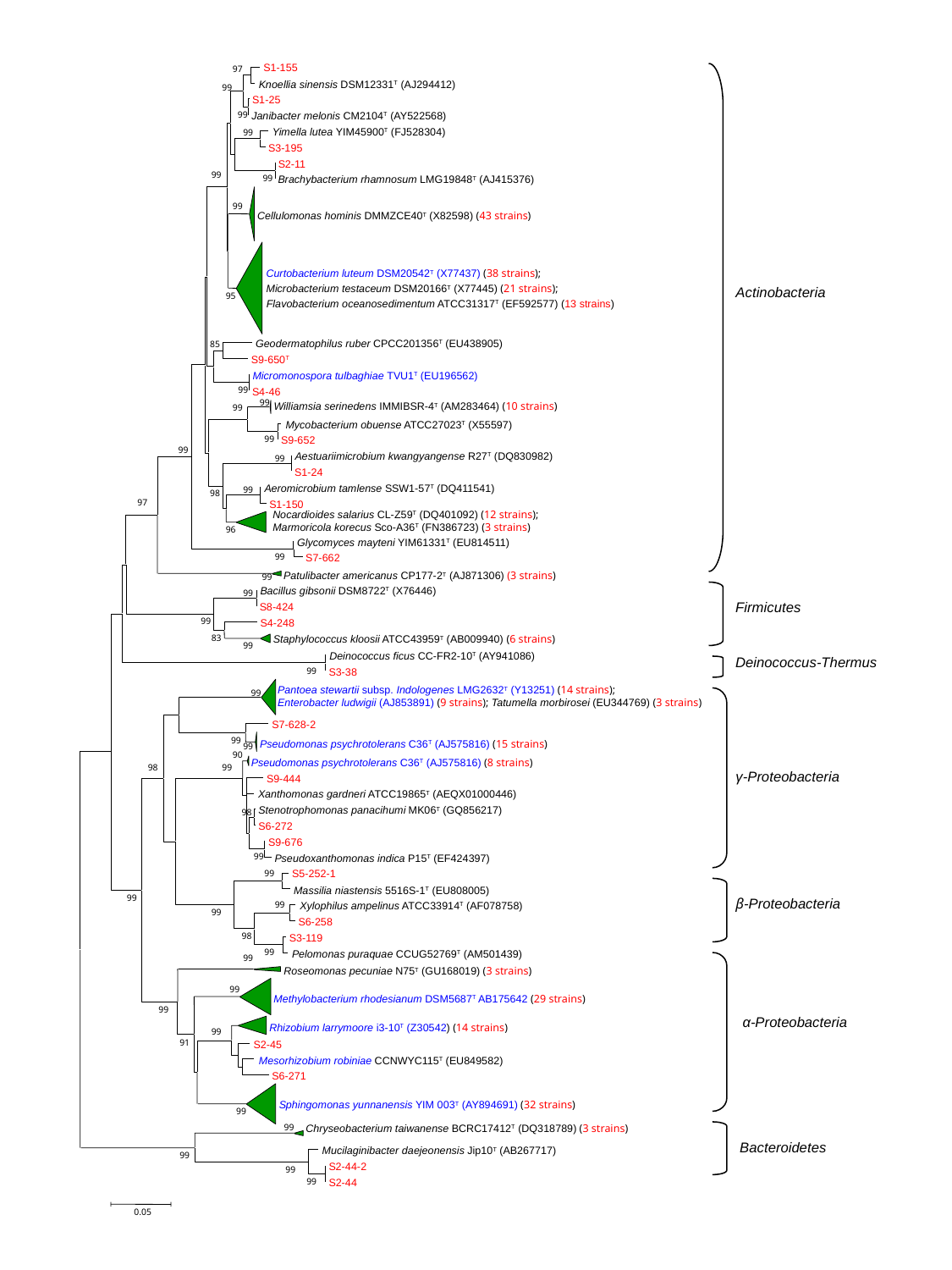

S1-155
97
 Knoellia sinensis DSM12331T (AJ294412)
99
 S1-25
99
 Janibacter melonis CM2104T (AY522568)
 Yimella lutea YIM45900T (FJ528304)
99
 S3-195
 S2-11
99
99
 Brachybacterium rhamnosum LMG19848T (AJ415376)
99
 Cellulomonas hominis DMMZCE40T (X82598) (43 strains)
 Curtobacterium luteum DSM20542T (X77437) (38 strains);
 Microbacterium testaceum DSM20166T (X77445) (21 strains);
 Flavobacterium oceanosedimentum ATCC31317T (EF592577) (13 strains)
Actinobacteria
95
 Geodermatophilus ruber CPCC201356T (EU438905)
85
 S9-650T
 Micromonospora tulbaghiae TVU1T (EU196562)
99
 S4-46
99
 Williamsia serinedens IMMIBSR-4T (AM283464) (10 strains)
99
 Mycobacterium obuense ATCC27023T (X55597)
99
 S9-652
99
 Aestuariimicrobium kwangyangense R27T (DQ830982)
99
 S1-24
 Aeromicrobium tamlense SSW1-57T (DQ411541)
99
98
97
 S1-150
 Nocardioides salarius CL-Z59T (DQ401092) (12 strains);
 Marmoricola korecus Sco-A36T (FN386723) (3 strains)
96
 Glycomyces mayteni YIM61331T (EU814511)
99
 S7-662
 Patulibacter americanus CP177-2T (AJ871306) (3 strains)
99
 Bacillus gibsonii DSM8722T (X76446)
99
Firmicutes
 S8-424
99
 S4-248
83
 Staphylococcus kloosii ATCC43959T (AB009940) (6 strains)
99
Deinococcus-Thermus
 Deinococcus ficus CC-FR2-10T (AY941086)
99
 S3-38
 Pantoea stewartii subsp. Indologenes LMG2632T (Y13251) (14 strains);
 Enterobacter ludwigii (AJ853891) (9 strains); Tatumella morbirosei (EU344769) (3 strains)
99
 S7-628-2
99
 Pseudomonas psychrotolerans C36T (AJ575816) (15 strains)
99
90
 Pseudomonas psychrotolerans C36T (AJ575816) (8 strains)
98
99
γ-Proteobacteria
 S9-444
 Xanthomonas gardneri ATCC19865T (AEQX01000446)
 Stenotrophomonas panacihumi MK06T (GQ856217)
98
 S6-272
 S9-676
99
 Pseudoxanthomonas indica P15T (EF424397)
 S5-252-1
99
 Massilia niastensis 5516S-1T (EU808005)
β-Proteobacteria
99
 Xylophilus ampelinus ATCC33914T (AF078758)
99
99
 S6-258
98
 S3-119
99
 Pelomonas puraquae CCUG52769T (AM501439)
99
 Roseomonas pecuniae N75T (GU168019) (3 strains)
99
 Methylobacterium rhodesianum DSM5687T AB175642 (29 strains)
99
α-Proteobacteria
 Rhizobium larrymoore i3-10T (Z30542) (14 strains)
99
91
 S2-45
 Mesorhizobium robiniae CCNWYC115T (EU849582)
 S6-271
 Sphingomonas yunnanensis YIM 003T (AY894691) (32 strains)
99
99
 Chryseobacterium taiwanense BCRC17412T (DQ318789) (3 strains)
Bacteroidetes
 Mucilaginibacter daejeonensis Jip10T (AB267717)
99
 S2-44-2
99
99
 S2-44
0.05
