# Supplementary material for: Leaf-residing Methylobacterium species fix nitrogen and promote biomass and seed production in Jatropha curcas
Source: Biotechnol Biofuels. 2015 Dec 21;8:222. doi: 10.1186/s13068-015-0404-y (PMC4687150; doi:10.1186/s13068-015-0404-y)
Supplement: Supplementary file 6 — 10.1186/s13068-015-0404-y Phylogenetic positions of root endophytes. The tree was constructed based on the 16S rDNA sequences using the Neighbor-Joining method. Bootstrap values (using 1000 replicates) are indicated at the branching points. Scale bar represents % estimated substitutions. Candidate for nitrogen-fixers (shown in blue) indicate the presence of nifH gene as evidenced by PCR amplifications. The number of strains is shown in parenthesis in red. The yellow arrowhead sizes indicate the relative abundance of the genus. [file 13068_2015_404_MOESM6_ESM.pptx]

## Slide 1
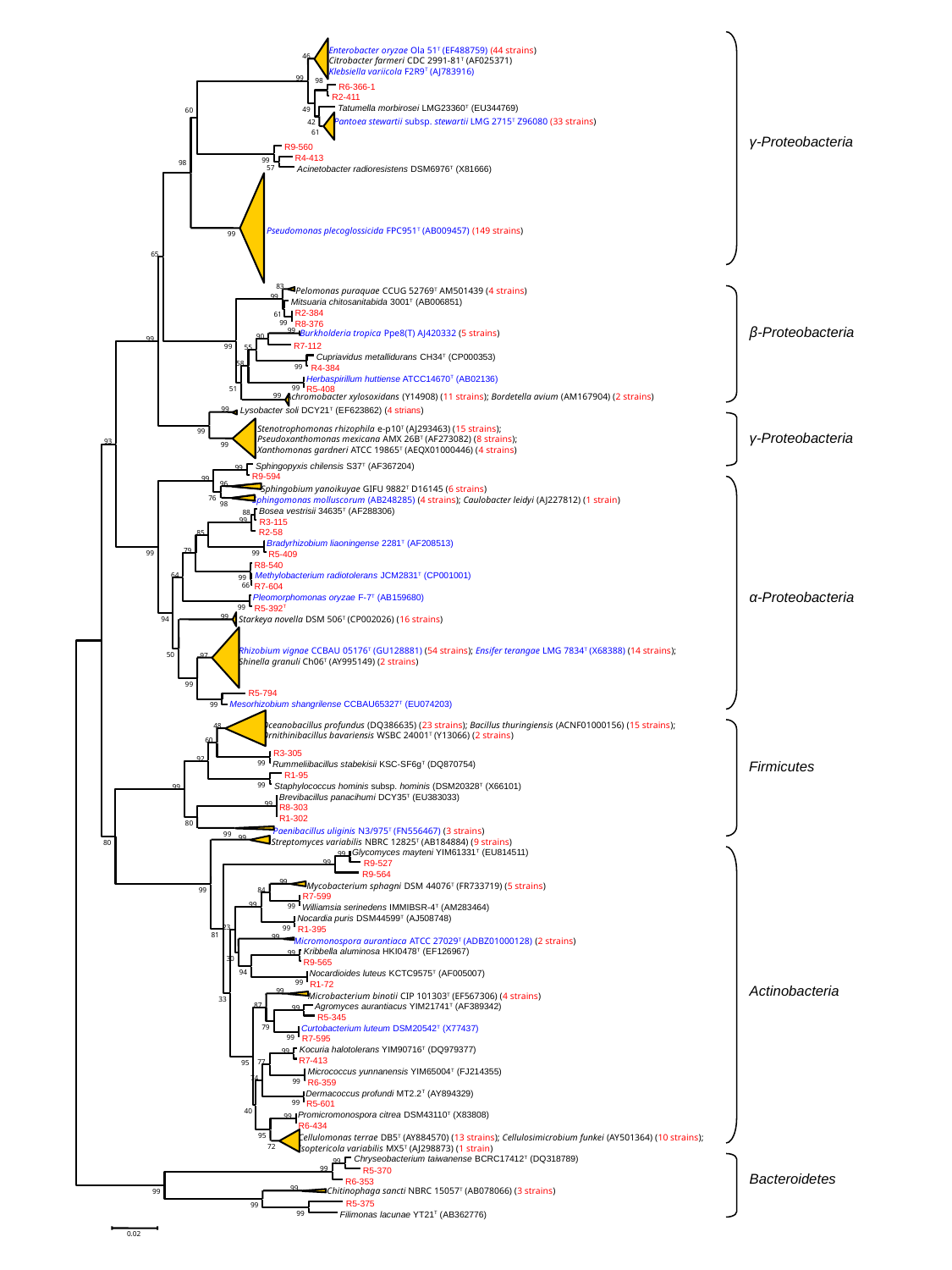

Enterobacter oryzae Ola 51T (EF488759) (44 strains)
 Citrobacter farmeri CDC 2991-81T (AF025371)
 Klebsiella variicola F2R9T (AJ783916)
46
99
98
 R6-366-1
 R2-411
 Tatumella morbirosei LMG23360T (EU344769)
49
60
 Pantoea stewartii subsp. stewartii LMG 2715T Z96080 (33 strains)
42
γ-Proteobacteria
61
 R9-560
 R4-413
99
98
57
 Acinetobacter radioresistens DSM6976T (X81666)
 Pseudomonas plecoglossicida FPC951T (AB009457) (149 strains)
99
65
83
 Pelomonas puraquae CCUG 52769T AM501439 (4 strains)
99
 Mitsuaria chitosanitabida 3001T (AB006851)
 R2-384
61
β-Proteobacteria
99
 R8-376
99
 Burkholderia tropica Ppe8(T) AJ420332 (5 strains)
90
99
 R7-112
99
55
 Cupriavidus metallidurans CH34T (CP000353)
58
99
 R4-384
 Herbaspirillum huttiense ATCC14670T (AB02136)
99
 R5-408
51
99
 Achromobacter xylosoxidans (Y14908) (11 strains); Bordetella avium (AM167904) (2 strains)
 Lysobacter soli DCY21T (EF623862) (4 strians)
99
 Stenotrophomonas rhizophila e-p10T (AJ293463) (15 strains);
 Pseudoxanthomonas mexicana AMX 26BT (AF273082) (8 strains);
 Xanthomonas gardneri ATCC 19865T (AEQX01000446) (4 strains)
γ-Proteobacteria
99
93
99
 Sphingopyxis chilensis S37T (AF367204)
99
 R9-594
99
96
 Sphingobium yanoikuyae GIFU 9882T D16145 (6 strains)
76
 Sphingomonas molluscorum (AB248285) (4 strains); Caulobacter leidyi (AJ227812) (1 strain)
98
 Bosea vestrisii 34635T (AF288306)
88
99
 R3-115
 R2-58
85
 Bradyrhizobium liaoningense 2281T (AF208513)
79
99
99
 R5-409
 R8-540
 Methylobacterium radiotolerans JCM2831T (CP001001)
64
99
66
 R7-604
α-Proteobacteria
 Pleomorphomonas oryzae F-7T (AB159680)
99
 R5-392T
99
 Starkeya novella DSM 506T (CP002026) (16 strains)
94
 Rhizobium vignae CCBAU 05176T (GU128881) (54 strains); Ensifer terangae LMG 7834T (X68388) (14 strains);
 Shinella granuli Ch06T (AY995149) (2 strains)
50
97
99
 R5-794
 Mesorhizobium shangrilense CCBAU65327T (EU074203)
99
 Oceanobacillus profundus (DQ386635) (23 strains); Bacillus thuringiensis (ACNF01000156) (15 strains);
 Ornithinibacillus bavariensis WSBC 24001T (Y13066) (2 strains)
48
60
 R3-305
Firmicutes
92
99
 Rummeliibacillus stabekisii KSC-SF6gT (DQ870754)
 R1-95
99
 Staphylococcus hominis subsp. hominis (DSM20328T (X66101)
99
 Brevibacillus panacihumi DCY35T (EU383033)
99
 R8-303
 R1-302
80
 Paenibacillus uliginis N3/975T (FN556467) (3 strains)
99
99
 Streptomyces variabilis NBRC 12825T (AB184884) (9 strains)
80
 Glycomyces mayteni YIM61331T (EU814511)
99
99
 R9-527
 R9-564
99
 Mycobacterium sphagni DSM 44076T (FR733719) (5 strains)
84
99
 R7-599
99
99
 Williamsia serinedens IMMIBSR-4T (AM283464)
 Nocardia puris DSM44599T (AJ508748)
23
99
 R1-395
81
99
 Micromonospora aurantiaca ATCC 27029T (ADBZ01000128) (2 strains)
 Kribbella aluminosa HKI0478T (EF126967)
99
30
 R9-565
94
 Nocardioides luteus KCTC9575T (AF005007)
Actinobacteria
99
 R1-72
99
 Microbacterium binotii CIP 101303T (EF567306) (4 strains)
33
87
 Agromyces aurantiacus YIM21741T (AF389342)
99
 R5-345
79
 Curtobacterium luteum DSM20542T (X77437)
99
 R7-595
 Kocuria halotolerans YIM90716T (DQ979377)
99
 R7-413
77
95
 Micrococcus yunnanensis YIM65004T (FJ214355)
74
99
 R6-359
 Dermacoccus profundi MT2.2T (AY894329)
99
 R5-601
40
 Promicromonospora citrea DSM43110T (X83808)
99
 R6-434
95
 Cellulomonas terrae DB5T (AY884570) (13 strains); Cellulosimicrobium funkei (AY501364) (10 strains);
 Isoptericola variabilis MX5T (AJ298873) (1 strain)
72
 Chryseobacterium taiwanense BCRC17412T (DQ318789)
99
99
Bacteroidetes
 R5-370
 R6-353
99
 Chitinophaga sancti NBRC 15057T (AB078066) (3 strains)
99
 R5-375
99
99
 Filimonas lacunae YT21T (AB362776)
0.02
