# Supplementary material for: Leaf-residing Methylobacterium species fix nitrogen and promote biomass and seed production in Jatropha curcas
Source: Biotechnol Biofuels. 2015 Dec 21;8:222. doi: 10.1186/s13068-015-0404-y (PMC4687150; doi:10.1186/s13068-015-0404-y)
Supplement: Supplementary file 7 — 10.1186/s13068-015-0404-y Cultivable endophytic bacterial diversity of Jatropha. Distribution of the phylotypes (%) over the different phyla and classes. The bar of each phylogenetic group is subdivided according to the different identification levels of the phylotypes. The group “identified at species level” contains those phylotypes that belong to existing species with a 16S rDNA homology threshold of ≥ 99.0 %. The group “identified at genus level” contains phylotypes that, based on phylogeny, belong to a particular genus and may represent a new species within that particular genus or an existing species within this genus showing < 99 % 16S rRNA gene sequence pairwise similarity with the type strain of this species. The group “potential gen. nov.” contains those phylotypes that could not be assigned to a particular genus (< 97 % pairwise similarity) based on the phylogeny of the 16S rRNA gene. [file 13068_2015_404_MOESM7_ESM.pptx]

## Slide 1
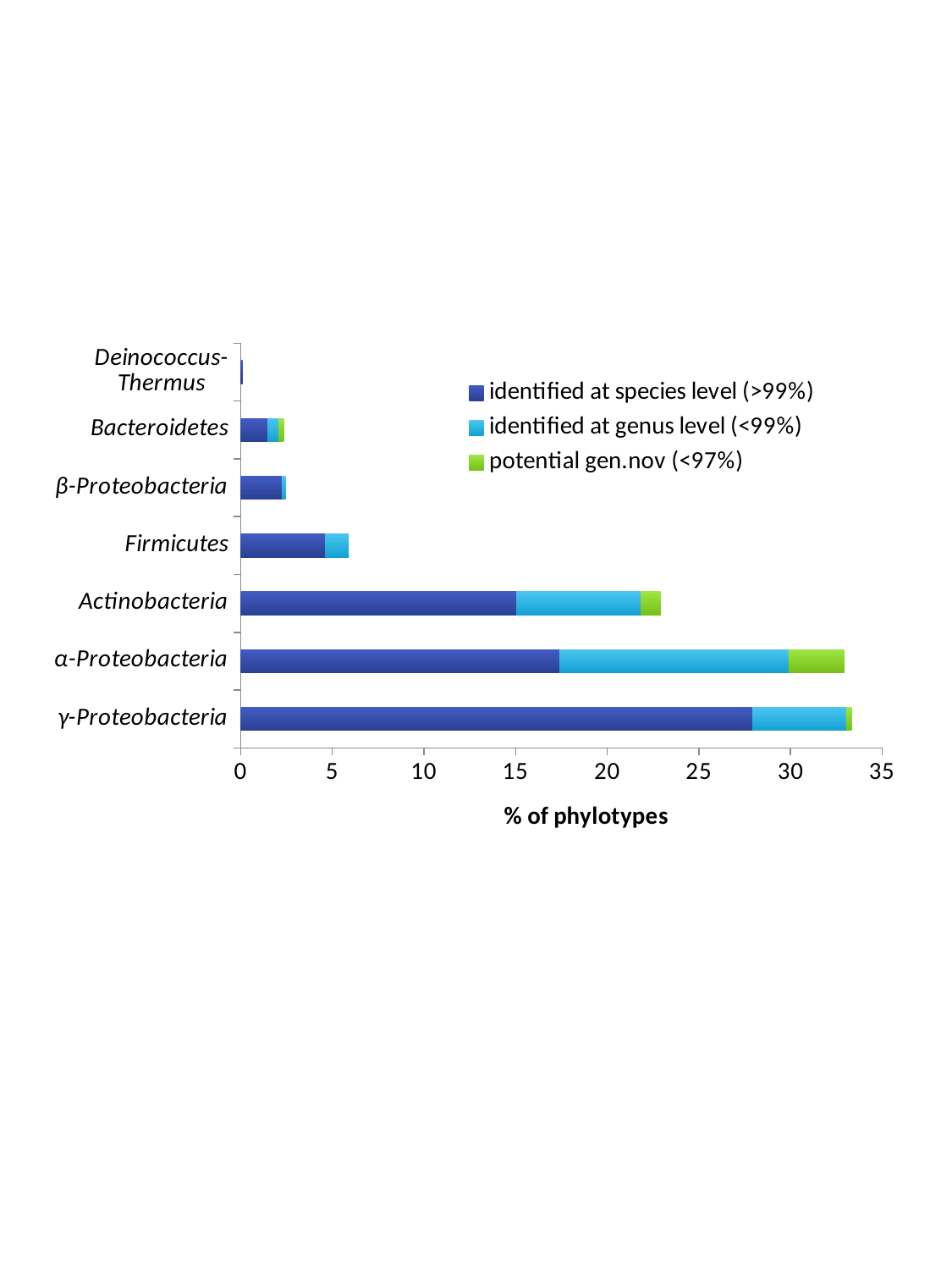

### Chart
| Category | | | |
|---|---|---|---|
| γ-Proteobacteria | 27.92527040314651 | 5.113077679449336 | 0.294985250737465 |
| α-Proteobacteria | 17.40412979351029 | 12.48770894788595 | 3.04818092428712 |
| Actinobacteria | 15.04424778761054 | 6.784660766961652 | 1.081612586037365 |
| Firmicutes | 4.621435594886888 | 1.27826941986234 | 0.0 |
| β-Proteobacteria | 2.261553588987232 | 0.196656833824975 | 0.0 |
| Bacteroidetes | 1.474926253687304 | 0.589970501474926 | 0.294985250737465 |
| Deinococcus-Thermus | 0.0983284169124881 | 0.0 | 0.0 |
